# Deficiency of *Fam20b*-Catalyzed Glycosaminoglycan Chain Synthesis in Neural Crest Leads to Cleft Palate

**DOI:** 10.3390/ijms24119634

**Published:** 2023-06-01

**Authors:** Xiaoyan Chen, Nan Li, Ping Hu, Leilei Li, Danya Li, Han Liu, Lei Zhu, Jing Xiao, Chao Liu

**Affiliations:** 1 Department of Oral Pathology, School of Stomatology, Dalian Medical University, Dalian 116044, China; 2 Dalian Key Laboratory of Basic Research in Oral Medicine, School of Stomatology, Dalian Medical University, Dalian 116044, China

**Keywords:** *Fam20b*, proteoglycans, glycosaminoglycan chains, cleft palate, elevation, osteogenesis

## Abstract

Cleft palate is one of the most common birth defects. Previous studies revealed that multiple factors, including impaired intracellular or intercellular signals, and incoordination of oral organs led to cleft palate, but were little concerned about the contribution of the extracellular matrix (ECM) during palatogenesis. Proteoglycans (PGs) are one of the important macromolecules in the ECM. They exert biological functions through one or more glycosaminoglycan (GAG) chains attached to core proteins. The family with sequence similarity 20 member b (Fam20b) are newly identified kinase-phosphorylating xylose residues that promote the correct assembly of the tetrasaccharide linkage region by creating a premise for GAG chain elongation. In this study, we explored the function of GAG chains in palate development through *Wnt1-Cre; Fam20b^f/f^* mice, which exhibited complete cleft palate, malformed tongue, and micrognathia. In contrast, *Osr2-Cre; Fam20b^f/f^* mice, in which *Fam20b* was deleted only in palatal mesenchyme, showed no abnormality, suggesting that failed palatal elevation in *Wnt1-Cre; Fam20b^f/f^* mice was secondary to micrognathia. In addition, the reduced GAG chains promoted the apoptosis of palatal cells, primarily resulting in reduced cell density and decreased palatal volume. The suppressed BMP signaling and reduced mineralization indicated an impaired osteogenesis of palatine, which could be rescued partially by constitutively active *Bmpr1a*. Together, our study highlighted the key role of GAG chains in palate morphogenesis.

## 1. Introduction

Cleft palate is the most common congenital malformations caused by genetic mutations or environmental stimulus [1,2]. The mammalian palate is composed of cells derived from cranial neural crest cells (CNCCs) and epithelial cells derived from pharyngeal ectoderm [3]. In mice, the palatal process grew vertically on either side of the tongue in the common oral–nasal cavity at early stage. Then, the palatal shelves are re-oriented horizontally above the tongue by overcoming the tongue obstruction, and eventually contacting with each other along the midline to fuse into a complete palatal floor covering the top of the oral cavity [3]. Cleft palate is caused by intrinsic factors, such as elevation or fusion abnormalities, or by disharmony in oral structure, such as physical obstruction of the tongue. There has been a significant amount of evidence of impaired intercellular or intracellular signals and coordination of oral structures leading to cleft palate [4,5,6]. Inactivation of *Pax9*, *Gli2,* or *Osr2* in palatal mesenchyme impairs palatal elevation, while the loss of *Msx1* and *Lhx8*, and the conditional ablation of *Tgfbr2* in palatal neural crest suppresses palatal growth [7,8,9,10,11]. Moreover, the mice with homozygous null for *Irf6* developed cleft palate via inappropriate oral adhesions and palatal fusion [12,13]. In contrast, little is known about the contribution of the extracellular matrix (ECM) to palate development. Thus, the role of the ECM during palatogenesis, especially in the horizontal elevation of palatal shelves, has attracted increasing attention.

As one of the most important biomacromolecules in ECM, proteoglycans (PGs) are found in intracellular, pericellular, and intercellular spaces, and participate in cell signaling, morphogenesis, cell migration, proliferation, differentiation, and apoptosis through their negatively charged glycosaminoglycan (GAG) chains [14,15]. According to the repetitive disaccharide units in the GAG chains, PGs are categorized into four types: heparan sulfate (HS), chondroitin sulfate (CS)/dermatan sulfate (DS), keratan sulfate (KS), and hyaluronic acid (HA) [15,16,17]. Although different from one another, the GAG chains of PGs are attached to core proteins through a common tetrasaccharide linker (GlcUAβ1-3Galβ1-3Galβ1-4Xylβ1-O-Ser). The conserved tetrasaccharide region passes through the corresponding specific glycosyltransferase (xylosyltransferase (XylT), galactosyltransferase I, (GalT-I), galactosyltransferase II (GalT-II), and β1,3-glucuronyltransferase I (β 1,3-glucuronyltransferase I, GlcAT-I)) successively adding monosaccharide residues to form a long GAG chain [18,19]. In this process, transient phosphorylation of xylose residues is an important modification that promotes the correct assembly of the tetrasaccharide linkage region, which functions as a molecular switch to regulate subsequent GAG chain assembly [20,21]. Therefore, the transient phosphorylation of xylose residues is not only a prerequisite for a complete tetrasaccharide linkage region, but also a key step for the elongation of GAG chains.

The family with sequence similarity to 20 member b (Fam20b) are newly identified kinase-phosphorylating xylose residues in the common linker tetrasaccharide region, that increase the activity of galactosyltransferase II (GalT-II), an enzyme necessary for linkage region and glycosaminoglycan assembly [22,23,24]. *Fam20b* knockout mice exhibited severe stunting and increased mortality at E13.5 embryos [25]. Inactivation of *Fam20b* in the oral epithelium led to supernumerary incisors and compromised enamel [26,27], while deletion of *Fam20b* by *Col1-cre* led to severe spine deformity and intervertebral disc disorder via the altered P38, ERK, and JNK signaling pathways [28]. Previously, we reported that *Wnt1-Cre; Fam20b^f/f^* mice, in which the *Fam20b*-catalyzed synthesis of GAG chains in CNCC-derived mesenchyme was disrupted, displayed a variety of craniofacial malformations, including cleft palates, microcephaly, micrognathia, cranial suture widening, and decreased mineralization of the cranio–maxillofacial bone and temporomandibular joint [29]. All of these findings indicated that *Fam20b*-catalyzed glycosylation plays a crucial role in craniofacial morphogenesis and mineralization.

CSPGs, DSPGs, HSPGs, and HA are all enriched in developing palatal shelves [30,31,32,33]. The GAG chains, predominantly HA, which are accumulated in palatal mesenchyme and combined a large number of water molecules, are considered as the main driving force for palatal expansion and elevation. However, conditional knockout hyaluronic acid synthase 2 (*Has2*) in *Wnt1-cre* mice resulted in micrognathia and secondary cleft palate, while *Osr2-Cre; Has2^f/f^* only caused a delay in palatal elevation, denying that the elastic force of GAG chains drives palatal elevation [34,35]. It is noteworthy that inactivation of *Golgb1*, a Golgi-associated large transmembrane protein, led to a cleft palate with reduced HA accumulation and impaired protein glycosylation in palatal mesenchyme [36], implicating that the GAG chains other than HA play a role in palatal development. In this study, to examine if the complete cleft palate in *Wnt1-Cre; Fam20b^f/f^* mice resulted from the primary defects in palatal mesenchyme, we first checked the capacity of palatal elevating. Then, to further explore the influence of *Fam20b* inactivation to palatal development, the cell survival, gene expression, and osteogenesis in *Wnt1-Cre; Fam20b^f/f^* palatal mesenchyme were evaluated.

## 2. Results

### 2.1. The Reduced GAG Content in the Palatal Mesenchyme of Wnt1-Cre; Fam20b^f/f^ Mice

**Figure 1:**
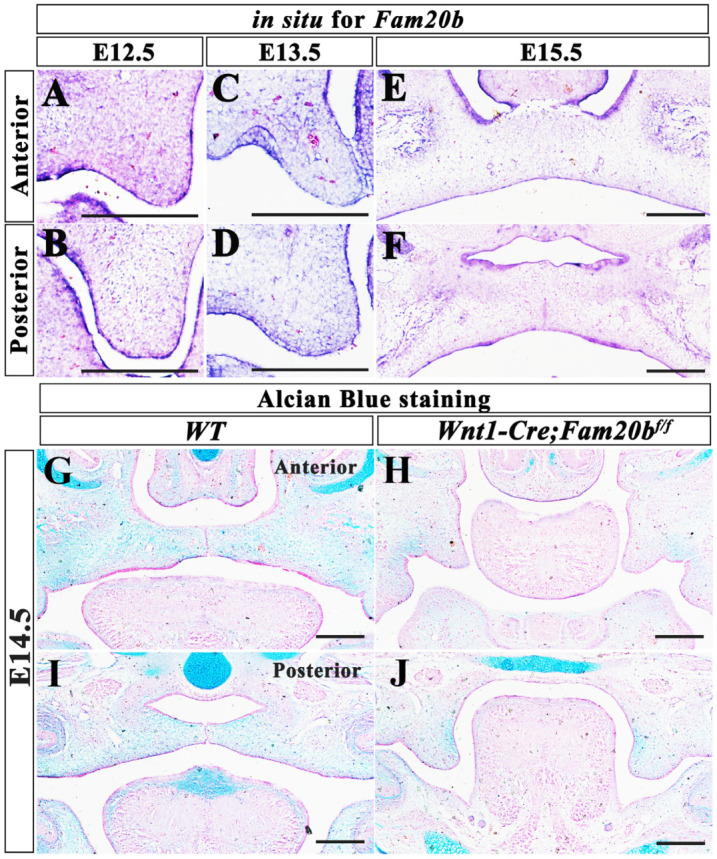
*Fam20b* expression pattern and GAG chain content in the developing *Wnt1-Cre; Fam20b^f/f^* palates. (**A**–**F**) In situ hybridization of *Fam20b* in wild-type mice palate at E12.5 (**A**,**B**), E13.5 (**C**,**D**), and E15.5 (**E**,**F**). (**G**–**J**) Alcian blue staining of E14.5 anterior part of palate (**G**,**H**) and posterior part of palate (**I**,**J**) in wild-type (**G**,**I**), and *Wnt1-Cre; Fam20b^f/f^* embryos (**H**,**J**). Scale bars, 200 μm.

We first examined the expression pattern of *Fam20b* by in situ hybridization in wild-type mice. *Fam20b* was detectable in both epithelium and mesenchyme in the E12.5 and E13.5 palatal shelves (Figure 1A–D). At E12.5, *Fam20b* was expressed uniformly in both anterior and posterior palatal mesenchyme (Figure 1A,B), while at E13.5, it was concentrated in the center of the anterior palate and the mesenchyme underlying the epithelium in the posterior palate (Figure 1C,D). At E15.5, the *Fam20b* expression domain was restricted to the epithelium and ossifying center (Figure 1E,F).

To explore the effect of *Fam20b* inactivation on GAG chain content, Alcian blue staining was performed. We found that the intensity of Alcian blue staining were apparently reduced in E14.5 *Wnt1-Cre; Fam20b^f/f^* palatal shelves, tongue, nasal cartilage, and Meckel’s cartilage comparing with wild-type littermates (Figure 1G–J), suggesting decreased GAG chain accumulation in CNCC-derived organs. More importantly, *Wnt1-Cre; Fam20b^f/f^* mice exhibited complete cleft palate after E14.5 (Figure 1G–J). The lateral palatal processes of *Wnt1-Cre; Fam20b^f/f^* were significantly smaller than those of the wild-type littermates, and failed to elevate horizontally at E14.5 (Figure 1G–J).

### 2.2. Wnt1-Cre; Fam20b^f/f^ Palatal Shelves Were Capable of Elevating

**Figure 2:**
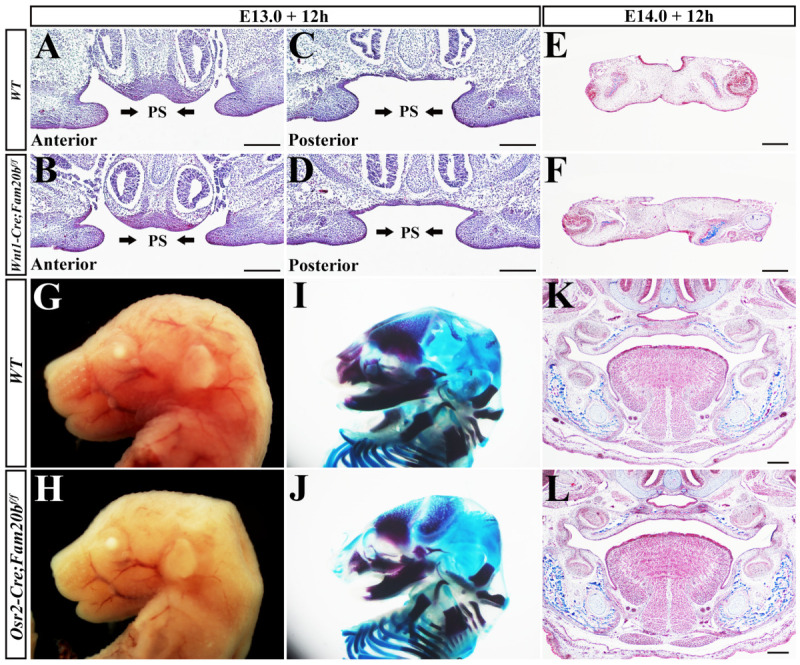
Organ culture of *Wnt1-Cre; Fam20b^f/f^* palatal shelves and the craniofacial morphology of *Osr2-Cre; Fam20b^f/f^* mice. (**A**–**D**) Masson staining of the anterior (**A**,**B**) and posterior (**C**,**D**) palatal shelves from E13.0 wild-type (**A**,**C**) and *Wnt1-Cre; Fam20b^f/f^* (**B**,**D**) heads without mandible and tongue after 12 h organ culture. (**E**,**F**) Masson staining showed the palatal shelves from both E14.0 wild-type (**E**) and *Wnt1-Cre; Fam20b^f/f^* (**F**) mice were fused after 12 h organ culture. (**G**,**H**) Sagittal views of E16.5 wild-type and *Osr2-Cre; Fam20b^f/f^* heads. (**I**,**J**) Alcian blue–Alizarin red staining of E16.5 wild-type and *Osr2-Cre; Fam20b^f/f^* embryos. (**K**,**L**) Masson staining of coronal sections of E16.5 wild type and *Osr2-Cre; Fam20b^f/f^*. Arrows indicate the orientation of palatal shelves. PS, palatal shelves; T, tongue; Th, tooth; MC, Meckel’s cartilage; scale bars, 200 μm.

To examine if the complete cleft palate in *Wnt1-Cre; Fam20b^f/f^* mice resulted from the primary defects in palatal mesenchyme, we performed organ culture ex vivo to examine the elevating capacity of *Wnt1-Cre; Fam20b^f/f^* palatal shelves. In the E13.0 *Wnt1-Cre; Fam20b^f/f^* mouse heads without tongue and mandible, both the anterior and posterior palatal shelves were able to elevate in the ex vivo organ culture (Figure 2A–D). Moreover, the E14.0 *Wnt1-Cre; Fam20b^f/f^* mouse palatal shelves fused with each other, as the WT controls did in the ex vivo organ culture (Figure 2E,F), suggesting that *Wnt1-Cre; Fam20b^f/f^* palatal shelves also retained the ability to fuse. Therefore, the cleft palate or the failed palatal elevation in *Wnt1-Cre; Fam20b^f/f^* mice was supposed to be a secondary deformity. We further generated *Osr2-Cre; Fam20b^f/f^* mice to examine this conclusion. Worthy of noticing, *Osr2-Cre; Fam20b^f/f^* mice showed intact palates, as well as little-affected tongues and mandibles compared with the wild-type counterparts (Figure 2G–L). Taken together, these results demonstrated that *Fam20b* inactivation caused few primary defects in palatal elevation, and the failed palatal elevation in *Wnt1-Cre; Fam20b^f/f^* mice most likely resulted from the undescending tongue.

### 2.3. Wnt1-Cre; Fam20b^f/f^ Mice Exhibited Human Pierre-Robin-Sequence-Like Phenotype

**Figure 3:**
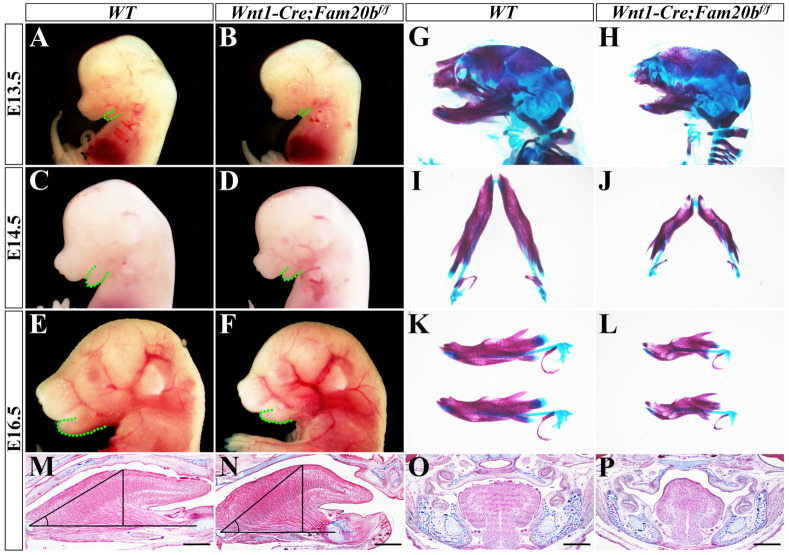
*Wnt1-Cre; Fam20b^f/f^* mice exhibited Pierre-Robin-sequence-like phenotype. (**A**–**F**) The gross lateral views of E13.5 (**A**,**B**), E14.5 (**C**,**D**), and E16.5 (**E**,**F**) wild-type and *Wnt1-Cre; Fam20b^f/f^* heads. The green dotted lines outlined mandibles in wild-type (**A**,**C**,**E**) and *Wnt1-Cre; Fam20b^f/f^* (**B**,**D**,**F**) embryos. (**G**–**L**) Alcian blue–Alizarin red staining of E16.5 wild-type and *Wnt1-Cre; Fam20b^f/f^* mandible. (**M**,**N**) Masson staining of sagittal sections of E16.5 wild-type and *Wnt1-Cre; Fam20b^f/f^* tongue to show the angle between the highest point of the tongue and the horizontal plane. (**O**,**P**) Masson staining of coronal sections of E16.5 wild-type and *Wnt1-Cre; Fam20b^f/f^* tongue. Scale bars, 500 μm.

*Wnt1-Cre; Fam20b^f/f^* new born die within 24 h after birth with cleft palates and micrognathia. Actually, from E13.5 on, the mandibles of *Wnt1-Cre; Fam20b^f/f^* mice started to be shorter and smaller than those of control mice, which became more and more severe in the following stages (Figure 3A–F). Bone and cartilage staining also confirmed the shortened mandibles of *Wnt1-Cre; Fam20b^f/f^* mice (Figure 3G–L). Moreover, the *Wnt1-Cre; Fam20b^f/f^* mandibular bones were excessively curved, which significantly increased the distal angle (Figure 3I,J). The sagittal view of *Wnt1-Cre; Fam20b^f/f^* mice showed that the angle between the highest point of the tongue and the horizontal plane increased significantly (32.67° ± 0.57° vs. 38.33° ± 1.52°, *p* < 0.01; Figure 3M,N), which led to the undescending tongue compared with wild-type control (Figure 3O,P). Since the micrognathia, undescending tongue, and cleft palates in *Wnt1-Cre; Fam20b^f/f^* mice highly resembled the human Pierre-Robin sequence [37,38,39], the failed elevation in *Wnt1-Cre; Fam20b^f/f^* palatal shelves was a consequence secondary to the shortened mandible.

### 2.4. Reduced Cell Density in Wnt1-Cre; Fam20b^f/f^ Palatal Mesenchyme

**Figure 4:**
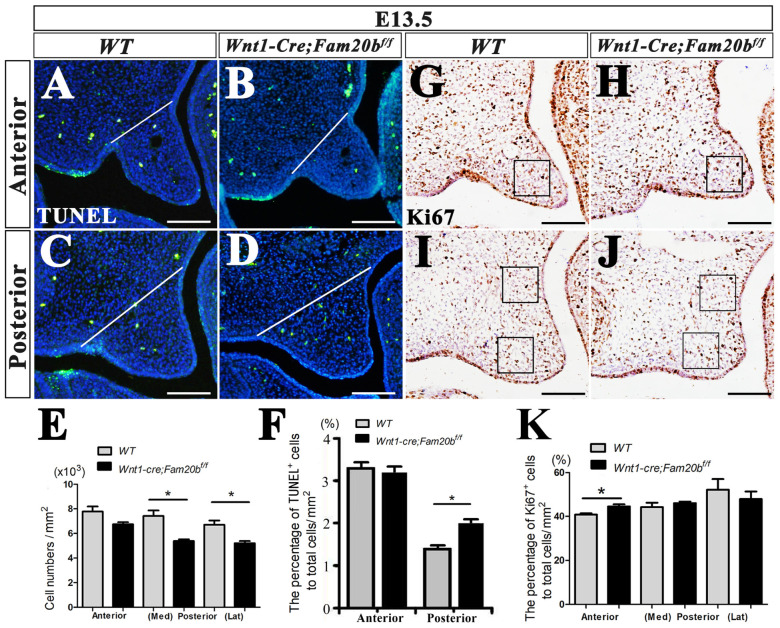
Cell density, apoptosis, and proliferation assays in *Wnt1-Cre; Fam20b^f/f^* palatal shelves. (**A**–**D**) TUNEL assay in the anterior (**A**,**B**) and posterior (**C**,**D**) E13.5 wild-type and *Wnt1-Cre; Fam20b^f/f^* palatal shelves. (**E**) Statistical assay of total mesenchymal cells in E13.5 wild-type and *Wnt1-Cre; Fam20b^f/f^* palates. (**F**) Statistical assay of the percentage of TUNEL positive cells to total cells in E13.5 wild-type and *Wnt1-Cre; Fam20b^f/f^* palatal shelves. (**G**–**J**) Ki67 immunohistochemical staining in the anterior (**G**,**H**) and posterior (**I**,**J**) E13.5 wild-type and *Wnt1-Cre; Fam20b^f/f^* palatal shelves. (**K**) Statistical assay of the percentage of Ki67 positive cells to total cells in restricted area of E13.5 wild-type and *Wnt1-Cre; Fam20b^f/f^* palatal shelves. Black box, restricted area of palatal shelves (1 mm^2^). *, *p* < 0.05. Scale bars, 100 μm.

Although the cleft palate in *Wnt1-Cre; Fam20b^f/f^* mice resulted secondarily from the Pierre-Robin-sequence-like phenotype, the primary defects in *Wnt1-Cre; Fam20b^f/f^* palates were not excluded. The size of *Wnt1-Cre; Fam20b^f/f^* palatal shelves was found to be significantly smaller than that in the control group. Statistical analysis of DAPI-stained nuclei showed that the number of total cells in restricted area of anterior palates showed no difference compared with wild type (anterior: 7800 ± 1300 cells/mm^2^ vs. 6780 ± 810 cells/mm^2^, *p* > 0.05), while the cell densities in the restricted medial and lateral regions of the E13.5 *Wnt1-Cre; Fam20b^f/f^* posterior palates were significantly reduced (medial: 7400 ± 1370 cells/mm^2^ vs. 5366 ± 633 cells/mm^2^, *p* < 0.05; lateral: 6700 ± 1060 cells/mm^2^ vs. 5200 ± 900 cells/mm^2^, *p* < 0.05; Figure 4A–E) Then, we investigated whether reduced cell density resulted from the altered cell proliferation and apoptosis in *Wnt1-Cre; Fam20b^f/f^* palatal shelves. TUNEL assay showed that the percentage of apoptotic cells in the total cells of posterior palate was significantly increased (anterior: 3.2987 ± 0.7942% vs. 3.2112 ± 0.8842%, *p* > 0.05; posterior: 1.3829 ± 0.8825% vs. 2.1457 ± 0.0842%, *p* < 0.05) (Figure 4A–D,F). In contrast, Ki67 staining displayed that the percentage of proliferating cells only in anterior palate was slightly increased (40.7733 ± 0.0118% vs. 44.5367 ± 0.0256%, *p* < 0.05), but there was no difference in posterior palates (medial: 44.2130 ± 0.3126% vs. 46.3123 ± 0.0176%, *p* > 0.05; lateral: 52.4143 ± 0.9846% vs. 47.9921 ± 0.6987%, *p* > 0.05; Figure 4G–K). In summary, the reduced size of *Wnt1-Cre; Fam20b^f/f^* palates resulted predominantly from the increased cell apoptosis and the decreased mesenchymal cell density, which most likely also led to cleft palates by disrupting palatal fusion, even in the case of accomplished palatal elevation.

### 2.5. The Altered Gene Expression in Wnt1-Cre; Fam20b^f/f^ Palatal Mesenchyme

**Figure 5:**
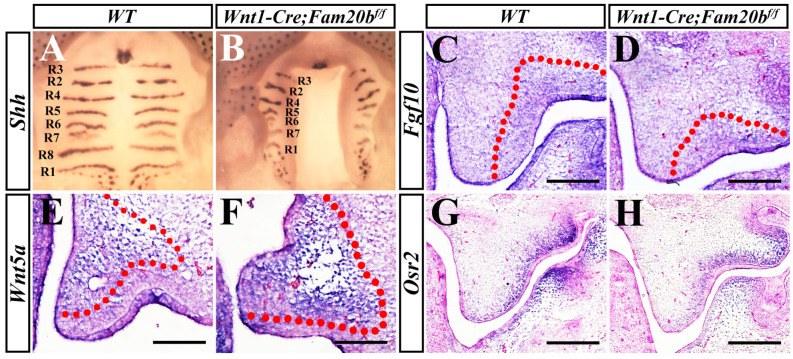
Altered gene expression pattern in *Wnt1-Cre; Fam20b^f/f^* palates. (**A**,**B**) Whole mount in situ hybridization showed the *Shh* expression pattern in E15.5 wild-type and *Wnt1-Cre; Fam20b^f/f^* palates. (**C**,**D**) *Fgf10* expression in E13.5 wild-type and *Wnt1-Cre; Fam20b^f/f^* posterior palatal shelves. (**E**,**F**) *Wnt5a* expression in E13.5 wild-type and *Wnt1-Cre; Fam20b^f/f^* anterior palatal shelves. (**G**,**H**) *Osr2* transcription in E13.5 wild-type and *Wnt1-Cre; Fam20b^f/f^* posterior palatal shelves. The red dashed line indicates the *Fgf10* and *Wnt5a* expression domains, respectively. Scale bars, 100 μm.

To further clarify the primary influence of GAG chains deficiency on palatal development, in situ hybridization was applied to examine gene expression pattern. The expression of *Shh* converged in the eight palatal rugae in wild-type mice at E15.5 (Figure 5A), while there was only seven rugaes in *Wnt1-Cre; Fam20b^f/f^* palates (Figure 5B). Moreover, the amount of *Shh* spots in *Wnt1-Cre; Fam20b^f/f^* soft palates was also significantly reduced compared to wild-type soft palates (Figure 5A,B). The *Fgf10* expression in the E13.5 *Wnt1-Cre; Fam20b^f/f^* posterior palatal mesenchyme was obviously decreased (Figure 5C,D). In contrast, *Wnt5a* transcription was enhanced in the E13.5 *Wnt1-Cre; Fam20b^f/f^* anterior palates (Figure 5E,F). Another transcription factor, *Osr2* which was specifically expressed in lateral mesenchyme of posterior palates, displayed an attenuated intensity in E13.5 *Wnt1-Cre; Fam20b^f/f^* posterior palates (Figure 5G,H). These altered gene expression patterns implicated a primary effect of GAG chain deficiency on palatal development.

### 2.6. The Palatine Osteogenesis in Wnt1-Cre; Fam20b^f/f^ Mice Was Impaired

**Figure 6:**
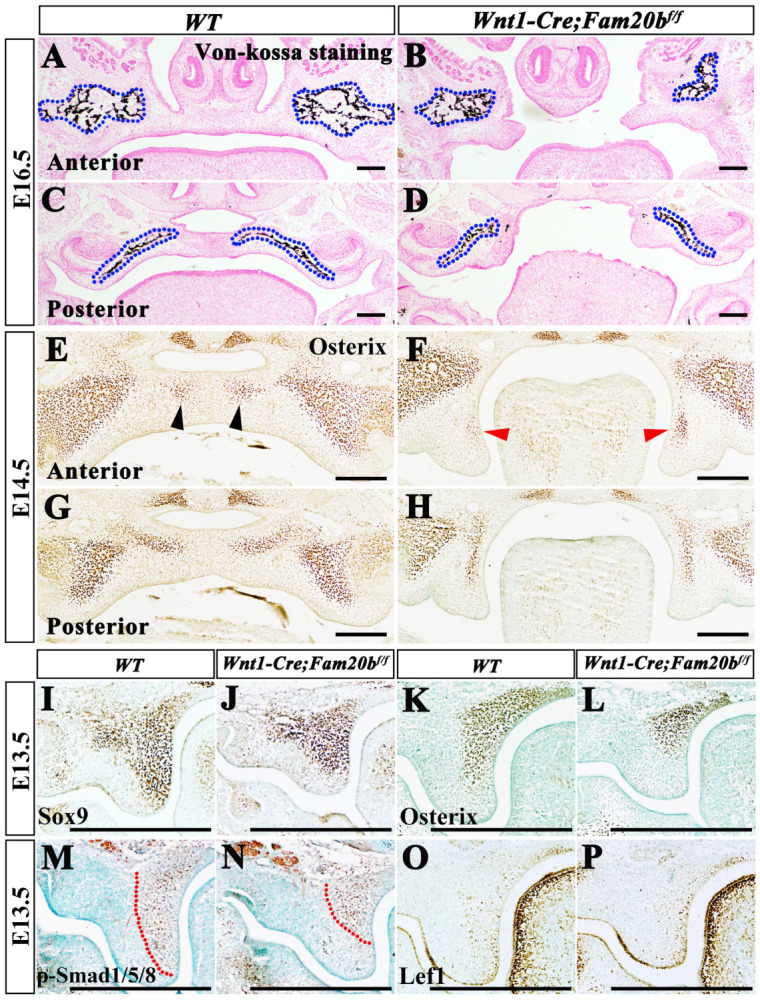
The altered osteogenic gene expression and signaling in *Wnt1-Cre; Fam20b^f/f^* palates. (**A**–**D**) Von Kossa staining in anterior (**A**,**B**) and posterior (**C**,**D**) palates in E16.5 wild-type and *Wnt1-Cre*; *Fam20b^f/f^* mice. The blue dashed lines in (**A**–**D**) outlined the ossifying palatines. (**E**–**H**) Immunohistochemistry staining of Osterix in anterior (**E**,**F**) and posterior (**G**,**H**) palates in E14.5 wild-type and *Wnt1-Cre; Fam20b^f/f^* mice. Black and red arrows indicate Osterix-positive cells in wild-type (**E**) and *Wnt1-Cre; Fam20b^f/f^* palates (**F**), respectively. (**I**–**L**) Immunohistochemistry staining of Sox9 (**I**,**J**) and Osterix (**K**,**L**) in E13.5 wild-type and *Wnt1-Cre; Fam20b^f/f^* palates. (**M**–**P**) Immunohistochemistry staining of p-Smad1/5/8 (**M**,**N**) and Lef1 (**O**,**P**) in E13.5 wild-type and *Wnt1-Cre; Fam20b^f/f^* palates. The red dashed lines delineate the p-Smad1/5/8 positive domains. Scale bars, 200 μm.

Since previous studies showed that deficient GAG chains synthesis affected primarily cartilage and bone development [40,41,42,43], we investigated the osteogenesis in *Wnt1-Cre; Fam20b^f/f^* palates. Von Kossa staining showed a significantly decreased mineral deposition in E16.5 *Wnt1-Cre; Fam20b^f/f^* palatine (Figure 6A–D). Consistently, the immunostaining of the key osteogenic marker, Osterix, showed a reduced expression domain and intensity in E14.5 *Wnt1-Cre; Fam20b^f/f^* palatal shelves (Figure 6E–H), suggesting a suppression of osteogenic differentiation of the secondary ossification center. To explore how GAG chains affected palatine osteogenesis, we examined the osteogenic gene expression and signaling. Both Sox9 (Figure 6I,J), a marker of the osteo–chondrogenic mesenchyme, and Osterix (Figure 6K,L), displayed the reduced domains in the lingual mesenchyme of E13.5 *Wnt1-Cre; Fam20b^f/f^* palates. Similar to the alteration of Sox9 and Osterix, the phosphorylated-Smad1/5/8 domain detected in the lingual mesenchyme of *Wnt1-Cre; Fam20b^f/f^* posterior palate was noticeable reduced compared with wild-type mice (Figure 6M,N). In contrast, there was no difference in Lef1 staining between E13.5 *Wnt1-Cre; Fam20b^f/f^* and wild-type palatal shelves (Figure 6O,P). These results implicated that the deficient GAG chain synthesis impaired osteogenic differentiation by suppressing canonical BMP signaling.

### 2.7. Enhanced BMP Activity in Wnt1-Cre; Fam20b^f/f^ Mice Partially Rescued Micrognathia, Instead of Cleft Palate

**Figure 7:**
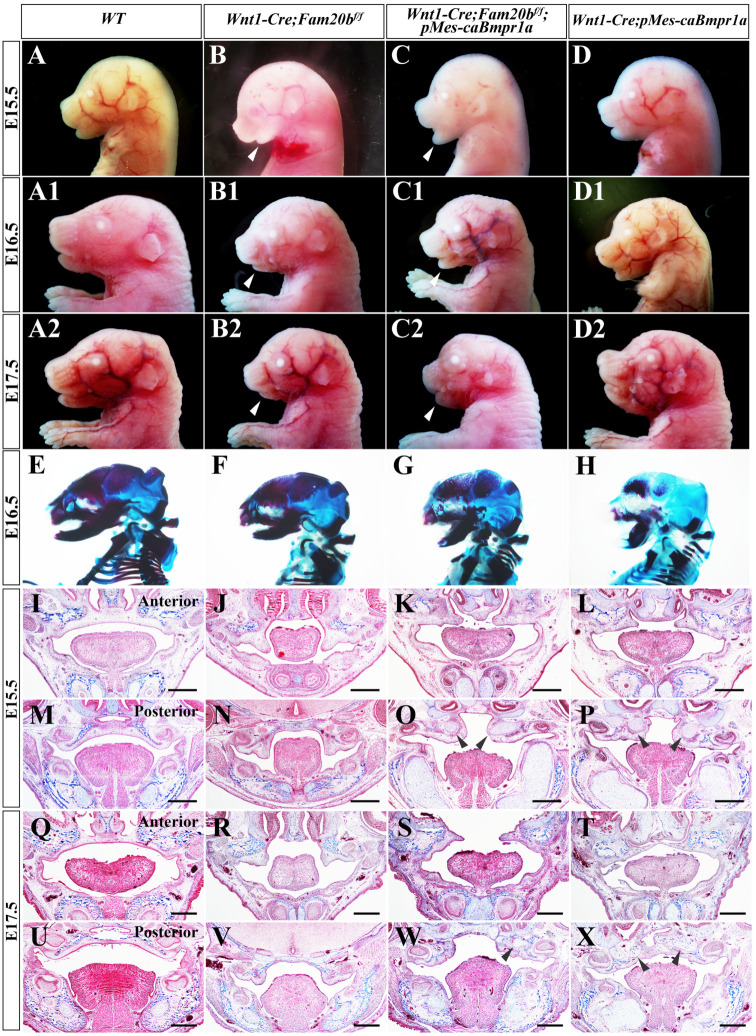
The craniofacial malformations of *Wnt1-Cre; Fam20b^f/f^* mice were partially rescued by the constitutively activated *Bmpr1a* transgene. (**A**–**D2**) The sagittal views of E15.5 (**A**–**D**), E16.5 (**A1**–**D1**) and E17.5 (**A2**–**D2**) wild-type (**A**,**A1**,**A2**), *Wnt1-Cre; Fam20b^f/f^* (**B**,**B1**,**B2**), *Wnt1-Cre; Fam20b^f/f^*; *pMes-caBmpr1a* (**C**,**C1**,**C2**), and *Wnt1-Cre; pMes-caBmpr1a* mouse heads (**D**,**D1**,**D2**). The white arrowheads point to the shortened mandibles in *Wnt1-Cre; Fam20b^f/f^* (**B**,**B1**,**B2**) and *Wnt1-Cre; Fam20b^f/f^*; *pMes-caBmpr1a* mice (**C**,**C1**,**C2**). (**E**–**H**) Alcian blue–Alizarin red staining of E16.5 wild type, *Wnt1-Cre; Fam20b^f/f^*, *Wnt1-Cre; Fam20b^f/f^*; *pMes-caBmpr1a,* and *Wnt1-Cre; pMes-caBmpr1a* mouse heads. (**I**–**X**) Masson staining of coronal sections of E15.5 (**I**–**P**) and E17.5 (**Q**–**X**) anterior and posterior palate of wild-type (**I**,**M**,**Q**,**U**), *Wnt1-Cre; Fam20b^f/f^* (**J**,**N**,**R**,**V**), *Wnt1-Cre; Fam20b^f/f^*; *pMes-caBmpr1a* (**K**,**O**,**S**,**W**), and *Wnt1-Cre; pMes-caBmpr1a* (**L**,**P**,**T**,**X**) mice. The black arrowheads point to the ectopic cartilages in *Wnt1-Cre; Fam20b^f/f^*; *pMes-caBmpr1a* (**O**,**W**) and *Wnt1-Cre; pMes-caBmpr1a* (**P**,**X**) palates. Scale bars, 500 μm.

To investigate the contribution of the suppressed canonical BMP signaling to *Wnt1-Cre; Fam20b^f/f^* cleft palate, we constitutively activated *Bmpr1a* in *Wnt1-Cre; Fam20b^f/f^* mice. The gross sagittal views showed that although still smaller than those of wild-type and *Wnt1-Cre; pMes-caBmpr1a* mice (Figure 7A–A2,D–D2), *Wnt1-Cre; Fam20b^f/f^*; *pMes-caBmpr1a* mandibles were significantly enlarged compared with *Wnt1-Cre; Fam20b^f/f^* mandibles (Figure 7B–B2,C–C2), implying a partially rescued micrognathia. Alcian blue–Alizarin red staining further confirmed that the *Wnt1-Cre; Fam20b^f/f^*; *pMes-caBmpr1a* mandibles were longer than *Wnt1-Cre; Fam20b^f/f^* mandibles (Figure 7E–H).

Histological staining showed that both *Wnt1-Cre; Fam20b^f/f^* and *Wnt1-Cre; pMes-caBmpr1a* mice had cleft palate, while the *pMes-caBmpr1a transgene (Wnt1-Cre; Fam20b^f/f^*; *pMes-caBmpr1a* mice) failed to rescue the cleft palates (Figure 7I–X). However, both E15.5 *Wnt1-Cre; Fam20b^f/f^*; *pMes-caBmpr1a* palatal shelves were located above the tongue, suggesting the palatal elevation was recovered by the enlarged mandibles. Although the *Wnt1-Cre; Fam20b^f/f^*; *pMes-caBmpr1a* palatal shelves were still too small to fuse into an intact palate (Figure 7K,O,S,W), the ectopic cartilages detected in the posterior palates and mandibles of both *Wnt1-Cre; Fam20b^f/f^*; *pMes-caBmpr1a* and *Wnt1-Cre; pMes-caBmpr1a* mice (Figure 7O,P,W,X) implied that the smaller palatal shelves most likely resulted from the enhanced BMP signaling, instead of completely from GAG chain deficiency. Actually, *Wnt1-Cre; Fam20b^f/f^*; *pMes-caBmpr1a* mice showed a milder chondrogenesis in palates and mandibles than *Wnt1-Cre; pMes-caBmpr1a* mice, implying the *Fam20b*-catalyzed GAG chain synthesis played a role opposite to BMP signaling in chondrogenesis (Figure 7K,L,O,P,S,T,W,X).

## 3. Discussion

### 3.1. The Deficient Fam20b-Catalyzed Synthesis of GAG Chains Impaired Mouse Craniofacial Development

*Fam20b*, as a member of secretory pathway kinases, is a newly identified hexokinase whose activity is a key switch in the biosynthesis of a wide array of O-linked PGs. *Fam20b*-dependent xylose phosphorylation in GAG chain synthesis has been demonstrated in vitro [22,23,44]. Inactivation of *Fam20b* in oral epithelium led to supernumerary incisors, highlighting the pivotal role of GAG chains in regulating the tooth number by determining the fate of dental epithelial stem/progenitor cells [26,27]. Loss of *Fam20b* in mouse cartilage led to chondrosarcoma in the knee joint and abnormal bone biomineralization [42], which was consistent with the results in zebrafish wherein inactivation of *Fam20b* led to aberrant organization of cartilage matrix and skeletal defects [43]; both were linked to abnormal GAG chain biosynthesis. In this study, we investigated the function of *Fam20b*-dependent GAG chains in craniofacial neural crest cells. Consistent with previous studies [42,43], the GAG chain accumulation in CNC-derived cells was significantly reduced, indicating the deficient synthesis of GAG chains due to *Fam20b* deletion. *Wnt1-Cre; Fam20b^f/f^* mice died of complete cleft palate within 24 h of birth. Except for cleft palate, the *Wnt1-Cre; Fam20b^f/f^* mice also exhibited microcephaly, widened fontanels, micrognathia, tongue elevation, temporomandibular joint abnormalities, and reduced mineralization in the skull, maxilla, mandible, and palatine. These results confirmed the essential role of *Fam20b* in craniofacial morphogenesis and mineralization. PGs are widely distributed in extracellular areas, the pericellular–basement membrane, cell surfaces, and intracellular spaces [17]. They interact with various signals, including BMP, WNT, FGF, and HH, mainly through different types of GAG chains [14,45]. Considering that inactivation of *Fam20b* affected the synthesis of several types of GAG chains, such as CS, DS, and HS, the impacts of knocking out *Fam20b* in CNC-derived cells on maxillofacial development could be diverse. In addition, the intracellular signaling pathways and intercellular communication affected by *Fam20b* loss would also be various. Therefore, further investigations are required to elucidate the specific mechanisms underlying the various craniofacial deformities caused by the inactivation of *Fam20b.*

### 3.2. The Primary and Secondary Effects of GAG Chains in Palatal Mesenchyme on Palatogenesis

The molecular mechanism controlling the elevation of the palatal shelves are still unclear. It was widely speculated that the anterior palate elevates in a ‘flip-up’ manner by the elastic force generated by the ECM, while the posterior palatal process remodels itself through regression in distal shelves and outgrowth in the horizontal direction [3]. Due to the capacity of binding a large amounts of water molecules to provide tissue plasticity, GAG chains were considered as the main source of elastic force for elevation [3,46]. In this study, *Wnt1-Cre; Fam20b^f/f^* mice exhibited a phenotype highly similar to human Pierre Robin sequence, which was characterized by a primary mandible malformation leading to tongue elevation and secondary cleft palate [37,38,39]. To verify whether elevation failure was secondary to micrognathia, we generated *Osr2-Cre; Fam20b^f/f^* mice, in which *Fam20b* was deleted only in the palatal mesenchyme, but not in mandible [47]. Since the *Osr2-Cre; Fam20b^f/f^* palate, tongue, and mandible exhibited no defect, the failed elevation in *Wnt1-Cre; Fam20b^f/f^* palates was caused by physical obstruction of tongue malposition rather than the loss of elastic force due to GAG chain deficiency. Furthermore, in vitro organ cultures directly confirmed that the palatal elevation and fusion were little affected by GAG chain deficiency. These results were consistent with those in *Wnt1-Cre; Has2^f/f^* mice [34,35], in which HA was eliminated, overthrowing the putative hypothesis that GAG chains generated the main driving force for palatal elevation.

Our results also showed that GAG chain deficiency leads to an apparent increase in apoptosis in the middle and posterior plate, and a reduced cell density, which caused a smaller palatal process, disabling the contact and fusion of palatal shelves. These results were contrary to the *Wnt1-Cre; Has2^f/f^* and *Golgb1* mutant mice, in which reduced ECM was accompanied with an increased cell density in palate mesenchyme [34,35,36]. Overall, our study indicated that the deficiency of *Fam20b*-catalyzed GAG chain synthesis not only resulted in secondary cleft palate by causing human Pierre-Robin-sequence-like phenotype, but also primarily suppressed cell survival and osteogenesis of palatal mesenchyme.

### 3.3. GAG Chains of Proteoglycans Were Involved in CNCC Osteogenesis by Mediating BMP Signaling

The palatine bone, as well as the mandibular bone, are directly differentiated from CNC-derived cells through intramembrane ossification [48,49,50]. In this study, the deficient synthesis of GAG chains resulted in micrognathia and abnormal palatine osteogenesis. Several signaling pathways were involved in osteoblast induction [48,49,50], among which the BMP pathway played essential roles in mesenchyme condensation and osteogenic differentiation during palatal osteogenesis [51]. We found a significant down-regulation of BMP signaling in *Wnt1-Cre; Fam20b^f/f^* palatal mesenchyme. The essential osteogenic transcription factors, including Sox9 and Osterix (Sp7), were also apparently reduced. The consistent results in *Osr2-IresCre*;*Bmpr1a^f/f^* mice [51] indicated that the affected palatal osteogenesis in *Wnt1-Cre; Fam20b^f/f^* mice was mainly caused by the suppressed BMP signaling. Subsequently, by enhancing BMP activity in CNC-derived mesenchyme through *caBmprIa* transgene, we showed a significantly increased osteogenesis in the anterior palate. Although *Wnt1-Cre; Fam20b^f/f^*; *pMes-caBmpr1a* still had a cleft palate, the elevated palatal process could be attributed to the partially rescued micrognathia by activated BMP signaling, suggesting that the activity of BMP signaling was mediated by GAG chains in maxillofacial osteogenesis. We previously reported that constitutively activated *Bmpr1a* or suppressed BMP signaling by over-expressing *Noggin* both resulted in cleft palate and abnormal osteogenesis [52], emphasizing the precise regulation of BMP signaling in palatal development. Currently, we hypothesized that inactivation of *Fam20b* led to a suppressed BMP signaling by attenuating signal enrichment or interactions between the growth factors and their receptors, but the specific mechanism still needed further explorations.

Currently, up to 27 skeletal disorders are reported to be associated with the mutations of 23 genes involved in GAG biosynthesis, which reflects the key roles of the GAG chains in the development and homeostasis of cartilage and bone [16,17]. Although *Fam20b* was indispensable in the osteogenesis and chondogenesis of zebra fish and mice, there were few reports on the function of *FAM20B* in humans. In the latest report, two lethal composite heterozygous variants in *FAM20B* (NM_014864 c.174_178delTACCT p.T59Afs*19/c.1038delG p.N347Mfs*4) were identified in a newborn who died soon after birth [41] and who suffered from a spectrum of defects highly similar to the autosomal recessive heterogeneous disorder, Desbuquois dysplasia (DBQD), which was characterized by joint laxity and skeletal change [53,54,55,56]. Several cases of DBQD have exhibited dysmorphic facial features, such as midface hypoplasia, flat nasal bridge, micrognathia, and cleft palate [57,58], resembling Pierre-Robin sequence and *Wnt1-Cre; Fam20b^f/f^* defects. Up to now, calcium-activated nucleotidase 1 gene (*CANT1*) and xylosyltransferase 1 (*XYLT1*) gene have been identified as the causative genes of DBQD [58,59]. Since both *CANT1* and *XYLT1* are involved in GAG chain synthesis, especially *XYLT1*, which functions upstream of *FAM20B* [41], *FAM20B* was most likely a novel causative gene for DBQD. Overall, our study provides new clues in the exploration of the pathogenesis and effective treatment of bone dysplasia, especially in craniofacial bones, resulting from deficient GAG chain synthesis.

## 4. Materials and Methods

### 4.1. Mouse Lines

All the animal experimental procedures in this study followed the protocol approved by the Animal Care and Use Committee at Dalian Medical University (Protocol No. AEE17038). The *Wnt1-Cre* mice, *Osr2-cre* knock-in mice (*Osr2-cre^KI^*), *Fam20b^f/f^* mice and *pMes-caBmpr1a* mice were described in previous studies [27,52,55,56]. To generate *Wnt1-Cre; Fam20b^f/f^* mice, we first crossed *Wnt1-Cre* mice with *Fam20b^f/f^* mice to obtain *Wnt1-Cre; Fam20b^f/+^* mice, and then, *Wnt1-Cre; Fam20b^f/+^* mice were mated with *Fam20b^f/f^* mice to generate *Wnt1-Cre; Fam20b^f/f^* mice. *Osr2-Cre; Fam20b^f/f^* mice was obtained in the same way. Genotyping was performed as previously described [27].

### 4.2. Alcian Blue Staining

The mouse embryonic heads were fixed overnight with 4% paraformaldehyde, dehydrated with gradient ethanol, embedded in paraffin, and made into 10 μm paraffin slices. The slices were stained with Alcian blue solution at pH 2.5 for 30 min, and counter-stained with nuclear fast red.

### 4.3. Skeleton and Cartilage Staining

The mouse embryos were fixed with anhydrous ethanol after removal of skin, viscera, and muscles, and then soaked in acetone to remove lipids. The working solution containing 0.1% Alcian blue and 0.3% Alizarin red was used for skeleton staining for 3 days, in which cartilage was stained blue and bone was stained red. Then, the excessively stained tissue was decolorized with 2% KOH. A stereoscopic microscope (Olympus, Shinjuku City, Japan) was used for imaging.

### 4.4. Organ Culture

The mandibles were removed from E13.0 and E14.0 embryonic heads, and collected in a 15 mL conical tube containing 3 mL DMEM, which was supplemented with 20% fetal calf serum (Invitrogen, Thermo Fisher Scientific, Waltham, MA, USA). Tubes were fixed in a roller incubator at 37 °C and 5% CO_2_ for 12 h culture. Then, the heads were washed in PBS and fixed in 4% paraformaldehyde for paraffin sections and histological staining.

### 4.5. Cell Proliferation and Apoptosis Analysis

Cell proliferation was detected by Ki67 immunostaining. After rehydration by gradient alcohol and antigen retrieval by citric acid, the paraffin sections were incubated with Ki67 (1:400) overnight at 4 °C. The Maxvision TM HRP polymer anti-mouse/rabbit IHC kit (Catalog No. KIT5020, Maixin Ltd., Shenzhen, China) was applied as a secondary antibody for 1 h incubation at room temperature, and then developed by 3,3′-diaminobenzidine (Catalog No. DAB-0031, Maixin Ltd., China) for coloring, and counterstained with hematoxylin. The apoptosis assay was conducted by In Situ Cell Death Detection Kit, POD (Catalog No. 11684817910, Roche, Basel, Switzerland) as instructed by manufacturer.

### 4.6. In Situ Hybridization

The mouse heads were fixed in paraformaldehyde treated with diethyl pyrocarbonate (DEPC), and then, dehydrated with gradient RNase free ethanol. Paraffin embedded heads were made into 10 um sections. Whole mounts and section in situ hybridization used digoxygenin-labelled antisense RNA probes (Catalog No. 11277073910, Roche, Switzerland) for *Fam20b*, *Shh*, *Fgf10, Wnt5a* and *Osr2* were followed by incubation with anti-digoxygenin-AP conjugate antibody (Catalog No. 11093274910, Roche, Switzerland). Bm-purple (Catalog No. 114420740010, Roche, Switzerland) was used for color development and nuclear fast red for counter-staining.

### 4.7. Immunohistochemistry

Gradient alcohol rehydration was performed on paraffin sections, and antigen repair was performed by citric acid repair solution with pH 6.0. The endogenous peroxidase and non-specific antigen binding sites were blocked by 3% H_2_O_2_ and 0.3% Triton X-100 sheep serum, respectively. Then, slides were incubated overnight at 4 °C with the primary antibodies against phosphorylated-Smad1/5/8 (Catalog No. 13820S, Cell Signaling Technology, Danvers, MA, USA), Sox9 (Catalog No. Ab185966, Abcam, Cambridge, UK) Osterix (Catalog No. Ab209484, Abcam, UK), and Lef1 (Catalog No. Ab137872, Abcam, UK), respectively. The second antibody was incubated at room temperature for one hour using MaxVision TM HRP Polymer anti-Mouse/Rabbit IHC Kit (Catalog No. KIT5020, Maixin Ltd., China), followed by 3,3′-Diaminobenzidine (Catalog No. DAB-0031, Maixin Ltd., China) color rendering and counter-stained with methyl green.

### 4.8. Statistical Assay

The experiments were performed with at least three biological replicates for statistical analysis in each group. Ki67 positive cells, TUNEL positive cells, and the total number of cells within the restricted areas were counted using Image J (version 1.46r, National Institutes of Health), followed by statistical analysis using GraphPad Prism 9 (GraphPad Software Inc., San Diego, CA, USA). Each group of data was analyzed using unpaired double-tailed Student’s *t*-test, and *p* value < 0.05 was considered statistically significant.

## Data Availability

The data of this study are available from the corresponding authors upon reasonable request.
